# Incorporation of dynamic segmented neutrophil-to-monocyte ratio with leukocyte count for sepsis risk stratification

**DOI:** 10.1038/s41598-019-56368-0

**Published:** 2019-12-24

**Authors:** Wen-Feng Fang, Yu-Mu Chen, Yi-Hsi Wang, Chi-Han Huang, Kai-Yin Hung, Ying-Tang Fang, Ya-Chun Chang, Chiung-Yu Lin, Ya-Ting Chang, Hung-Cheng Chen, Kuo-Tung Huang, Yun-Che Chen, Chin-Chou Wang, Meng-Chih Lin

**Affiliations:** 1 Division of Pulmonary and Critical Care Medicine, Department of Internal Medicine, Kaohsiung Chang Gung Memorial Hospital, Chang Gung University College of Medicine, Kaohsiung, Taiwan; 2 Department of Respiratory Therapy, Kaohsiung Chang Gung Memorial Hospital, Chang Gung University College of Medicine, Kaohsiung, Taiwan; 3 Department of Respiratory Care, Chang Gung University of Science and Technology, Chiayi, Taiwan; 4 Graduate Institute of Clinical Medical Sciences, Chang Gung University, Taoyuan, Taiwan; 5 Department of Nutritional Therapy, Kaohsiung Chang Gung Memorial Hospital, Kaohsiung, Taiwan

**Keywords:** Interleukins, Risk factors

## Abstract

The association between sepsis and segmented neutrophil-to-monocyte (SeMo) ratio is unclear. We postulated that an increase in dynamic SeMo ratio measurement can be applied in risk stratification. This retrospective study included 727 consecutive sepsis patients in medical intensive care units (ICUs), including a subpopulation of 153 patients. According to the leukocyte (white blood cell, WBC) count on day 3 (normal range, between 4,000/µL and 12,000/µL) and delta SeMo (value of SeMo ratio on day 3 minus value of SeMo ratio on day 1; normal delta SeMo, <7), patients were grouped into 3 (delta SeMo & WBC tool). The survival lines separated significantly with hazard ratios of 1.854 (1.342–2.560) for the delta SeMo or WBC abnormal group and 2.860 (1.849–4.439) for the delta SeMo and WBC abnormal group compared to the delta SeMo and WBC normal group. Delta SeMo & WBC tool and delta sequential organ failure assessment (SOFA) tool performed better than the other tools (delta SeMo, delta WBC, day 3 WBC, and day 1 WBC). Severity in delta SeMo & WBC tool and delta SeMo tool reflected the immune dysfunction score, cytokine expression, and human leukocyte antigen D-related monocyte expression on day 1 and day 3. There was correspondence between delta SOFA and delta WBC and between delta SeMo and delta cytokine expression. Incorporation of dynamic SeMo ratio with WBC count provides risk stratification for sepsis patients admitted in the ICU.

## Introduction

Sepsis is among the leading causes of mortality and morbidity^1^ in the intensive care units (ICUs). Sepsis patients suffer from life-threatening organ dysfunction due to a dysregulated host inflammatory response to infection^2^ and other factors such as endothelial dysfunction with interaction of leukocytes^3^. Because many factors other than the initial severity contribute to the mortality^4–6^ and patients usually present with different manifestations, many risk scores consist of several domains. However, simple dynamic tools for risk stratification are limited.

In sepsis, traditional systemic inflammatory variables include leukocytosis (white blood cell [WBC] count >12,000/µL) or leukopenia (WBC count <4,000/µL)^7^. Leukocytes consist of segmented neutrophils, lymphocytes, monocytes, and immature cells. During sepsis, initially, circulating neutrophils and monocytes respond to the pathogenic organisms^8^ as an immune defense mechanism. Activated monocytes release inflammatory cytokines in response to infection. Impaired cytokine production is a potential therapeutically modifiable surrogate endpoint^9^, but not clinically available.

The inflammatory cytokine overexpression is related to multiple system organ failure and mortality^10^. On the contrary, immunoparalysis, which is characterized by monocyte deactivation^11^, is also associated with poor clinical outcomes in sepsis. Our recent study showed that segmented neutrophil-to-monocyte (SeMo) ratio can help in predicting 28-day mortality in sepsis patients^12^ as one domain of immune dysfunction score. However, we had not investigated the application of delta SeMo ratio. We hypothesized that dynamic SeMo ratio measurement may provide information about the state of immune response in sepsis. We also postulated that the degree of increase in delta SeMo ratio measurement would be of incremental value in risk stratification. The relationship between delta SeMo ratio and multiple organ dysfunction is also unknown. Here, we demonstrate that delta SeMo ratio has a complementary role to WBC. These findings uncovered an important role of the tool integrating delta SeMo ratio and WBC in sepsis risk stratification. It represents the underlying modulation of inflammatory cytokines and immune response and organ dysfunction in sepsis and sheds light on the development of a simple marker to accomplish precision medicine for sepsis.

## Materials and Methods

### Setting

This was an analysis of data gathered in three medical ICUs (a total of 34 beds) from August 2013 to January 2017 at Kaohsiung Chang Gung Memorial Hospital, a 2,700-bed tertiary hospital in Southern Taiwan.

### Study design

The extension study was a post hoc analysis of an integrative research program, consisting of prospective observational investigation and retrospective medical record review^6,12–15^. This study retrospectively investigated the clinical factors (e.g., whole blood leukocyte count and dynamic SeMo ratio) in predicting outcomes in sepsis patients. Patients’ immune responses were also analyzed if data were available. All consecutive ICU patients who met the sepsis criteria (Sepsis-2) were initially screened. All those enrolled patients fulfilled the definition of sepsis (Sepsis-3)^12^. Analyzing white blood cell differentials (segmented neutrophils, eosinophils, lymphocytes, monocytes, and immature granulocytes) consisted of a cytochemical reaction of the cells with a reagent set (e.g., Lysercell WDF [organic quaternary ammonium salts 0.07%, nonionic surfactant 0.12%] and Fluorocell WDF [polymethine 0.002%, methanol 3.0%, ethylene glycol 96.9%] from Sysmex America), followed by fluorescence flow cytometric analysis by Sysmex XN-9000. SeMo ratio was calculated by dividing the mature segmented-neutrophil count by mature monocyte count. The study was approved by the Institutional Review Board of Chang Gung Memorial Hospital. We confirmed that all methods were performed in accordance with the relevant guidelines and regulations. In the subpopulation of patients who prospectively participated in immune profiling and cytokine analysis, patients or their surrogates signed the written informed consent.

### Data collection

**Figure 1:**
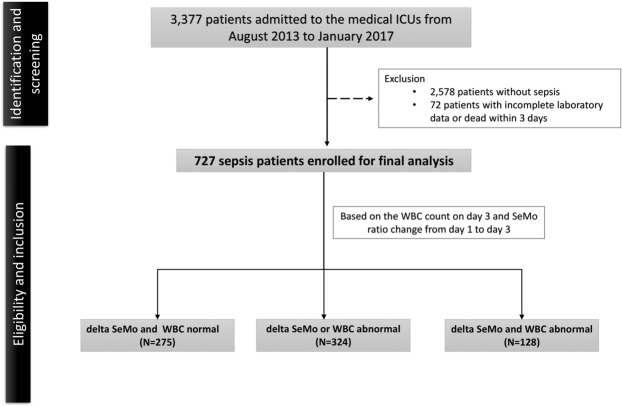
Flowchart. Abbreviation: SeMo = segmented neutrophil-to-monocyte ratio.

Clinical data were retrieved from medical records including complete blood cell count and differential count, Acute Physiology and Chronic Health Evaluation II (APACHE II) score, Charlson Comorbidity Index, sequential organ failure assessment (SOFA) score and underlying comorbidities, and other clinical factors. Moreover, a total of 153 out of the 727 patients (Fig. 1) had been enrolled in immune status and cytokine study with blood samplings performed based on the protocol on days 1 and 3 during ICU hospitalization.

### Immune status and cytokine study

Peripheral monocyte preparation and stimulation, measurements of human leukocyte antigen D-related (HLA-DR) monocyte expression, and cytokine levels in plasma or cell culture media have been described in our previous papers^12,13^.

### Statistical methods

As in our previous study, patient demographics, clinical characteristics, and outcomes are summarized using frequency and percentage for categorical variables and mean ± standard deviation for continuous variables. Differences between the groups (from different tools) were analyzed using Student’s t-test for continuous variables. Comparison analyses among the three groups (delta SeMo and WBC normal, delta SeMo or WBC abnormal, delta SeMo and WBC abnormal) were performed using Pearson chi-squared test and one-way analysis of variance as appropriate. Kaplan–Meier analysis was performed to test patient survival among groups. A receiver operating characteristic (ROC) curve and Youden’s index were used to determine the best cutoff values for 28-day mortality that were statistically significant in the univariate analysis. Statistical significance was set at a two-sided P value of <0.05. All data were analyzed using Statistical Package for the Social Sciences software version 22.0 (IBM Corp., Armonk, NY, USA). Area under ROC curve (AUR)comparison was performed using the DeLong method with MedCalc version 18.2.1.

### Ethics approval and consent to participate

The study was approved by the Institutional Review Board of Chang Gung Memorial Hospital. For the patients who prospectively participated in immune profiling and cytokine analysis, written informed consent was obtained from all the patients or their surrogates.

## Results

A total of 727 sepsis patients were enrolled for analysis (Fig. 1). Of them, 153 patients had immune profile study. In addition to higher APACHE II score, Charlson Comorbidity Index, and SOFA score, patients who died in 28 days had higher delta SeMo (value of SeMo ratio on day 3 minus value of SeMo ratio on day 1) (Table S1). Using ROC curve and Youden’s index, we determined that patients with delta SeMo ≥7 had poor clinical outcomes (Fig. S1).

### Comparison of different risk severity tools for mortality

**Figure 2:**
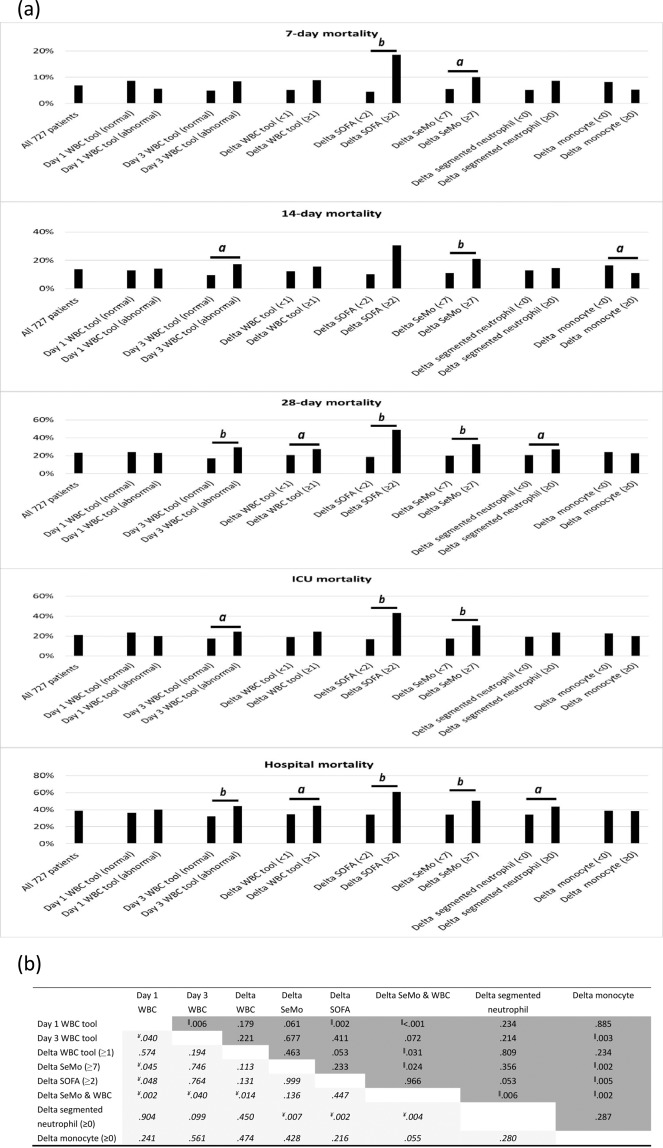
(**a**) Mortality by the tools in all sepsis patients. Mortality outcomes are summarized in percentage for variables using Pearson chi-squared test. Statistical significances show ***a*** as P value < 0.05 and ***b*** as P value < 0.001. (**b**) Comparison of ROC curve among the eight tools. Pairwise comparison of receiver operating characteristic curves (the number represents the P value); *The italicized cells represent the P value in pairwise comparison for predicting the 14-day mortality; the normal cells represent the P value for predicting the 28-day mortality; ^¥^Statistically significant difference in predicting 14-day mortality; ^||^ Statistically significant difference in predicting 28-day mortality.

We developed and tested tools for mortality risk stratification (Fig. 2(a)). Day 1 WBC count cannot differentiate survivors from non-survivors. Day 3 WBC count can differentiate 14-day, 28-day, ICU, and hospital mortality, but not 7-day mortality. If we take day 3 and day 1 data into account, delta SeMo, delta SOFA, and even delta WBC can be helpful for risk stratification. Tools using delta segmented-neutrophil and delta monocyte did not perform better than delta SeMo & WBC tool for risk stratification. Comparison of ROC curve among the eight tools is shown in Fig. 2(b). The delta SeMo & WBC tool and delta SOFA tool were better than the other tools (delta SeMo & WBC tool vs. delta SOFA tool, AUR = 0.62 vs. 0.62, P = 0.97). The delta SeMo & WBC tool was constructed as below.

### Construction of tool with the incorporation of dynamic segmented neutrophil-to-monocyte ratio with *leukocyte count (delta SeMo & WBC tool)*

According to the WBC count on day 3 and SeMo ratio change from day 1 to day 3, patients can be grouped into the following 3 groups: (1) the delta SeMo and WBC normal group (WBC count within the normal range between 4,000/µL and 12,000/µL and delta SeMo <7), (2) the delta SeMo or WBC abnormal group (WBC count on day 3 within the normal range between 4,000/µL and 12,000/µL but delta SeMo ≥7, or WBC count on day 3 not within normal range but delta SeMo <7), and (3) the delta SeMo and WBC abnormal group (delta SeMo ≥7 and WBC count on day 3 not within normal range).

### Application of delta SeMo & WBC tool

**Figure 3:**
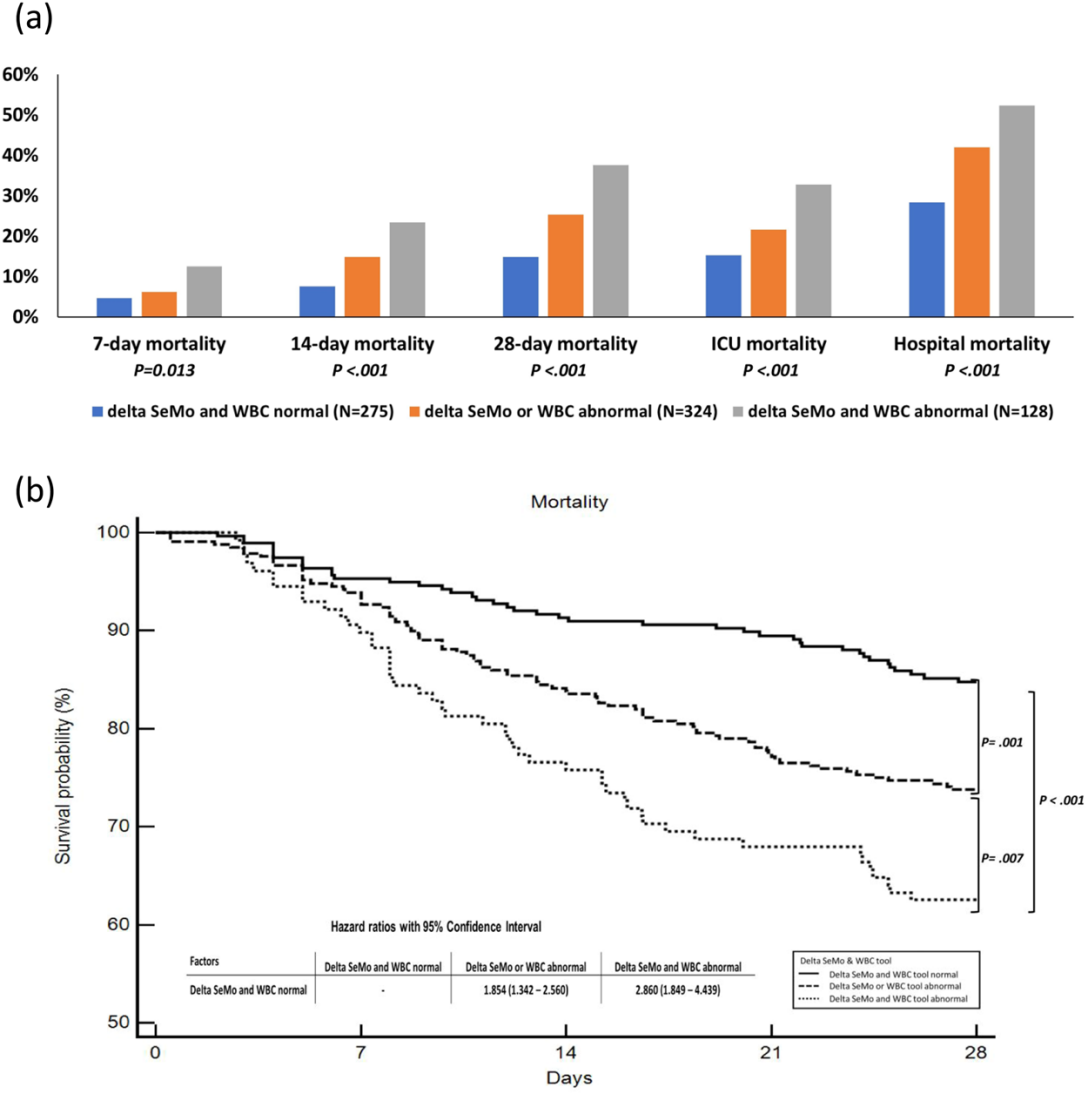
(**a**) Risk stratification by delta SeMo & WBC tool. (**b**) 28-day survival curve by the delta SeMo & WBC tool. Kaplan–Meier estimates of 28-day survival according to stratification by the SeMo & WBC tools in 727 sepsis patients.

At baseline, there were no significant differences among the three groups regarding age, gender, site of suspected infection, APACHE II score, Charlson Comorbidity Index, and SOFA score (Table 1). Using the risk stratification tool, we can group patients with different 7-day, 14-day, 28-day, ICU, and hospital survival rate (Fig. 3(a)). The survival lines separated significantly (Fig. 3(b)) with hazard ratios of 1.854 (1.342–2.560) for the delta SeMo or WBC abnormal group and 2.86 (1.849–4.439) for the delta SeMo and WBC abnormal group compared to the delta SeMo and WBC normal group.

**Table 1:** Baseline demographic and clinical characteristics of 727 sepsis patients stratified using the delta SeMo & WBC tool.

Demographics characteristics	delta SeMo and WBC normal (N = 275)	delta SeMo or WBC abnormal (N = 324)	delta SeMo and WBC abnormal (N = 128)	*P value*
Age, years	68.9 (14.6)	66.3 (15.4)	66.5 (14.1)	*0.082*
BMI, kg/m^2^	23.0 (4.6)	22.5 (4.9)	22.5 (5.6)	*0.358*
Sex, male (%)	161 (58.5)	199 (61.4)	64 (50.0)	*0.085*
Site of suspected infection, N (%)				
Pulmonary	181 (65.8)	204 (63.0)	85 (66.4)	*0.691*
Intra-abdominal	19 (6.9)	29 (9.0)	6 (4.7)	*0.273*
Urinary tract	70 (25.5)	69 (21.3)	23 (18.0)	*0.206*
Bacteremia	19 (6.9)	25 (7.7)	9 (7.0)	*0.924*
Unidentified infection	20 (7.3)	33 (10.2)	10 (7.8)	*0.420*
APACHE II score	23.8 (8.0)	24.1 (8.3)	25.5 (7.9)	*0.156*
Charlson Comorbidity Index	2.4 (1.8)	2.5 (1.9)	2.8 (2.1)	*0.114*
Coronary artery disease	81 (29.5)	73 (22.5)	31 (24.2)	*0.144*
Hypertension	161 (58.5)	181 (55.9)	70 (55.1)	*0.740*
COPD	39 (14.2)	48 (14.8)	22 (17.2)	*0.728*
Cancer	51 (18.8)	76 (23.6)	36 (28.3)	*0.087*
Chronic liver disease	36 (13.1)	41 (12.7)	15 (11.7)	*0.928*
Diabetes mellitus	123 (44.7)	141 (43.5)	63 (49.2)	*0.544*
History of stroke	55 (20.0)	56 (17.3)	25 (19.5)	*0.673*
Chronic kidney disease	85 (30.9)	93 (28.7)	43 (33.6)	*0.579*
**Day 1**				
SIRS	2.0 (1.0)	2.4 (0.9)	2.5 (1.0)	*<0.001*
q-SOFA	1.5 (0.6)	1.6 (0.7)	1.6 (0.7)	*0.235*
SOFA scores	8.4 (3.4)	8.9 (3.7)	9.2 (3.8)	*0.105*
White blood cell count	11,722.5 (5,882.4)	15,416.2 (9,145.8)	16,282.8 (8,643.1)	*<0.001*
Segmented neutrophil count	9,636.2 (5,229.9)	12,522.3 (8,274.9)	12,765.4 (7,507.7)	*<0.001*
Lymphocyte count	1,069.1 (930.0)	1,233.9 (1,556.6)	1,461.8 (1,532.6)	*0.023*
Monocyte count	512.6 (553.4)	637.4 (603.6)	842.8 (671.9)	*<0.001*
SeMo ratio	29.8 (24.7)	32.1 (39.7)	18.6 (15.6)	*<0.001*
C-reactive protein, mg/L	123.8 (109.3)	150.1 (115.6)	155.9 (119.8)	*0.014*
Lactate, mmol/L	24.2 (20.9)	33.7 (31.9)	33.1 (28.4)	*0.001*
Procalcitonin	19.9 (46.5)	24.4 (48.4)	29.3 (48.0)	*0.429*
I/O, fluid balance	308.9 (1165.3)	534.8 (1301.9)	710.9 (1307.9)	*0.007*

### Delta SeMo & WBC tool reflects sepsis severity and immune dysfunction

As shown in Table S2, there were significant differences among the three groups regarding systemic inflammatory response syndrome (SIRS), quick-SOFA (q-SOFA)^16^, and SOFA scores. Additionally, WBC count, differential counts, and SeMo ratio were also different. C-reactive protein level, lactate level, and input and output fluid balance were significantly lowest in the delta SeMo and WBC normal group. In a subpopulation of patients with immune status and cytokine data, we found that this stratification tool reflected the immune dysfunction score and G-CSF and HLA-DR expression levels (Table 2) with the advantage partly coming from delta SeMo tool showing different immune dysfunction scores, cytokines expressions, and HLA-DR expressions on day 1 and day 3 (Table 3). Moreover, some cytokines produced from monocytes stimulated with lipopolysaccharide (LPS) were different in the delta SeMo groups (Table S3).

**Table 2:** Immune status of 153 sepsis patients stratified by the delta SeMo & WBC tool.

Immune status (N = 153)	delta SeMo and WBC normal (N = 53)	delta SeMo or WBC abnormal (N = 69)	delta SeMo and WBC abnormal (N = 31)	*P value*
28-day mortality	9 (17.0)	28 (40.6)	12 (38.7)	*0.015*
**Day 1**				
Immune dysfunction score	1.2 (1.0)	1.7 (1.4)	2.1 (1.1)	*0.006*
SeMo ratio	29.2 (28.5)	37.7 (60.4)	17.5 (10.2)	*0.106*
G-CSF, pg/uL	225.4 (506.4)	1591.1 (4048.8)	716.0 (2917.6)	*0.046*
IL-10, pg/uL	32.3 (41.0)	120.1 (270.1)	95.2 (189.3)	*0.059*
HLA-DR expression %	92.2 (12.6)	85.0 (15.7)	81.1 (17.2)	*0.003*

G-CSF = granulocyte colony-stimulating factor; IL-10 = interleukin-10; HLA-DR = human leukocyte antigen-antigen D-related expression.

**Table 3:** Immune status of 153 sepsis patients stratified by the delta SeMo tool.

Immune status (N = 153)	delta SeMo <7 (N = 111)	delta SeMo ≥7 (N = 42)	*P value*
28-day mortality	13 (11.7)	11 (26.2)	*0.028*
**Day 1**			
Immune dysfunction score^**§**^	1.4 (1.2)	2.2 (1.3)	*<0.001*
SeMo ratio	35.4 (51.1)	18.2 (12.3)	*0.001*
Plasma			
G-CSF, pg/uL	676.3 (2,609.3)	1,639.4 (4,024.7)	*0.155*
IL-10, pg/uL	63.2 (173.3)	141.2 (264.5)	*0.082*
IL-17A, pg/uL	17.1 (33.9)	9.3 (19.5)	*0.079*
IL-1RA, pg/uL	577.4 (2223.0)	583.7 (1650.4)	*0.987*
IL-6, pg/uL	166.0 (339.9)	545.8 (2459.4)	*0.324*
TNF-α, pg/uL	57.9 (82.0)	85.5 (194.6)	*0.378*
VEGF, pg/uL	220.5 (446.1)	128.3 (199.0)	*0.199*
HLA-DR expression	88.3 (14.9)	82.4 (16.4)	*0.037*
**Day 3**			
Plasma	(N = 108)	(N = 40)	
G-CSF, pg/uL	303.6 (1739.6)	659.8 (1594.6)	*0.260*
IL-10, pg/uL	50.9 (186.7)	74.4 (180.8)	*0.494*
IL-17A, pg/uL	19.5 (38.9)	9.6 (18.6)	*0.041*
IL-1RA, pg/uL	333.3 (1421.7)	88.3 (143.9)	*0.280*
IL-6, pg/uL	105.8 (293.0)	117.6 (451.9)	*0.852*
TNF-α, pg/uL	47.7 (70.4)	52.5 (83.3)	*0.724*
VEGF, pg/uL	255.0 (574.1)	155.6 (271.4)	*0.295*
HLA-DR expression	91.0 (11.2)	82.9 (16.6)	*0.008*

^§^Immune dysfunction score.

G-CSF = granulocyte colony-stimulating factor; IL-10 = interleukin-10; IL-1RA = interleukin-1 receptor antagonist; IL-6 = interleukin-6; TNF-α = tumor necrosis factor-α; VEGF = vascular endothelial growth factor; HLA-DR = human leukocyte antigen-antigen D-related expression.

### Relationship between delta SeMo, delta sequential organ failure assessment, delta WBC, and delta cytokine expression

As the delta SeMo & WBC tool is composed of WBC count and delta SeMo, we assessed the association between delta SeMo, delta SOFA, delta WBC, and delta cytokine expression (Table S4). There was correspondence between delta SOFA and delta WBC. Furthermore, there were correspondences between delta SeMo and delta cytokine expression (inflammatory IL-6, anti-inflammatory IL-10).

## Discussion

The present study suggested the incorporation of dynamic SeMo ratio with leukocyte count as a simple and fast way for risk stratification in sepsis patients admitted in the ICU. This tool integrated the components of WBC and delta SeMo. Additionally, this tool has the following advantages: delta WBC correlates to delta SOFA and delta SeMo is associated with immune status and cytokine expression. This affordable tool can be easily applied in clinical practice without specific laboratory work condition for cytokine and monocyte HLA-DR expression assessment.

The complex immune system alterations seen during the onset of sepsis include pro-inflammatory, anti-inflammatory, and immunosuppressive responses as shown in our results. Recovery can be characterized by resolution of inflammation and recovery of immune cell paresis^9^. In sepsis patients, dysregulated host response to infection can be due to immunosuppression^17^ and potentially affects every organ system. Immunodeficiency is common and is a prognostic factor in sepsis patients^18^. Additionally, patients dying of sepsis may have marked immunosuppression induced by sepsis^19,20^. We can determine the immune system’s status through monocyte HLA-DR expression. Inhibition of HLA-DR expression with consequent deactivation of monocytes would facilitate the development and progression of sepsis^21^. In fact, some studies have shown that immunosuppression rather than inflammation is the major driving force for mortality in sepsis^19^. Another way of determining the immune system’s status is to assess the diminished capacity of monocytes from sepsis patients to release pro-inflammatory cytokines in response to endotoxin^22^. LPS is usually used as endotoxin to stimulate the monocytes^23^. Our study shows that delta SeMo ≥7 represents lower monocyte HLA-DR expression on day 1 and day 3 and lower survival rate. IL-17A^24^, which can induce the production of pro-inflammatory cytokines by monocytes/macrophages^25^, was also lower in the patient group with delta SeMo ≥7 on day 3. Using delta SeMo tool, we can find different cytokine expression profiles in serum or monocyte culture media stimulated with LPS or not. The complex cytokine expression network may mediate organ–organ cross talk and failure^26^; however, this is out of the scope of our present study. Interestingly, patients with delta SeMo ≥7 had poor immune dysfunction score^12^. Immune dysfunction score consists of HLA-DR expression, plasma G-CSF level, plasma IL-10 level, and SeMo ratio on day 1. Calculating delta SeMo score is much quicker and easier than calculating the immune dysfunction score. Although we did not focus on the investigation of immature granulocyte counts and their dynamic change, they appeared not to be useful in our population (Table S5).

Sepsis was defined as SIRS with suspected or confirmed infection. SIRS variables include body temperature, pulse rate, respiratory rate, and WBC count^27^. In our study, delta WBC is associated with delta SOFA. At first, SOFA score was designed to describe a sequence of complications, not to predict outcomes^28^. A previous study found that early changes in organ function, presented as delta SOFA, can help in predicting eventual survival in severe sepsis^29^. The dynamic tool is better than simple SOFA score on ICU admission. Multiple organ dysfunction is complicated and associated with organ–organ interaction^30,31^. In our study, we found that WBC count alone on day 1 cannot differentiate survival from non-survival. Since sepsis is a life-threatening organ dysfunction due to a dysregulated host response to infection, patients’ serial response is important. We assessed and compared delta WBC, delta SOFA, and delta SeMo. We found that delta SeMo & WBC tool was not inferior to delta SOFA tool regarding mortality prediction. Patient groups stratified by delta SeMo & WBC tool also represented increasing severity by SIRS, q-SOFA, and SOFA scores on day 3. Additionally, the levels of lactate, C-reactive protein, and fluid balance^32^ on day 1 and day 3 were also comparable with the severity and risk stratification. Moreover, calculating delta SeMo & WBC tool is quicker than calculating SOFA score.

In our previous prospective observational study^12^, we found that immune dysfunction score on day 1 can predict patients’ 28-day mortality. The immune dysfunction score consists of 4 components, including segmented neutrophil-to-monocyte ratio (SeMo ratio). Therefore, SeMo ratio attracted our attention. Although with day 3 and day 7 immune profile data, the previous study was limited by its relatively small number of patients for further analysis. We were also concerned about the possible selection bias during recruitment of immune profile study. Therefore, we retrospectively enrolled all consecutive ICU patients who met the sepsis criteria for analysis in this study. In this way, the above bias can be corrected for. The primary aim of the present study was to investigate the clinical factors (e.g., SeMo ratio) in predicting outcomes in sepsis patients. Patients’ immune responses and cytokines were also analyzed if data were available as an add-on advantage. We found that the SeMo & WBC tool performed well in total population and in subpopulation with immune profiles. As a new extension study, the study design was approved by the Institutional Review Board.

The strength of our study includes the findings of simple risk stratification tool, which reflects the complex organ dysfunction and immune status in consecutive sepsis patients. The analysis from a subpopulation of patients with immune profile relates the clinical tool to possible mechanism, which is immune dysfunction. However, we should apply this tool carefully since the study was conducted in sepsis patients admitted in the ICU and excluded patients who died within 3 days. With the absence of day 3 data, we cannot stratify those who died within 3 days using this tool. Although severe patients can be stratified using day 1 data to assess the immune dysfunction score as in our previous study, it is not affordable for clinical practice. Whether the tools can be applied in less severe sepsis patients treated out of the ICU is uncertain. Nevertheless, we are investigating the issues. Our data showed that the tool can also reflect SIRS, q-SOFA, and SOFA scores, making it applicable for patients in the ICU and out of the ICU^16,33,34^. Patients with cancer, chronic kidney disease, and cardiovascular disease were prone to higher mortality in our study population. We are analyzing whether these comorbidities are also related to immune dysfunction and poor survival in sepsis^14^. However, there are no significant differences in groups using delta SeMo & WBC tools regarding the above comorbidities.

## Conclusion

Incorporation of dynamic SeMo ratio with WBC count provides risk stratification for sepsis patients admitted in the ICU. The tool represents the underlying modulation of inflammatory cytokines and immune response in sepsis.

## Supplementary information

Supplementary information 

## Data Availability

The datasets used and/or analysed during the current study are available from the corresponding author on reasonable request.
