# Supplementary material for: Incorporation of dynamic segmented neutrophil-to-monocyte ratio with leukocyte count for sepsis risk stratification
Source: Sci Rep. 2019 Dec 24;9:19756. doi: 10.1038/s41598-019-56368-0 (PMC6930327; doi:10.1038/s41598-019-56368-0)
Supplement: Supplementary file 1 — Supplementary information [file 41598_2019_56368_MOESM1_ESM.pdf]

Supplemental Electronic Material

Incorporation of dynamic segmented neutrophil-to-monocyte ratio with leukocyte count for sepsis risk stratification

Authors

Wen-Feng Fang, MD; Yu-Mu Chen, MD; Yi-Hsi Wang, MD; Chi-Han Huang, BSc; Kai-Yin Hung, MSc; Ying-Tang Fang, MD; Ya-Chun Chang, MD; Chiung-Yu Lin, MD; Ya-Ting Chang, MSc; Hung-Cheng Chen, MD; Kuo-Tung Huang, MD; Yun-Che Chen, MD; Chin-Chou Wang, MD; Meng-Chih Lin, MD

Figure S1- ROC curve of the delta SeMo

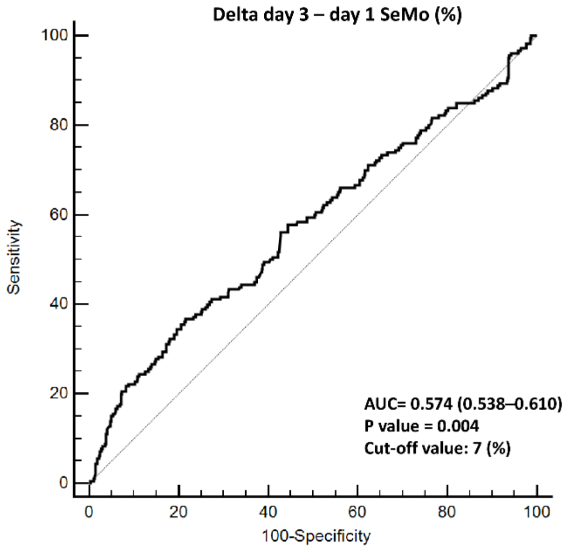

Figure legend

Receiver operating characteristic curve of the delta day 3–day 1 SeMo (%) for discrimination between 727 sepsis patients with 28-day mortality in the hospital

Table S1- Baseline characteristics of 727 sepsis patients

**Baseline characteristics**	**28-day survivor (N=556)**	**28-day non-survivor (N=180)**	**P value**
Sex, male	324 (58.3)	109 (60.6)	.589
APACHE II score	23.9 (8.3)	25.9 (7.4)	.005
Age, years	67.4 (15.0)	66.7 (14.6)	.536
BMI, kg/m^2	22.75 (5.0)	22.8 (4.6)	.899
Charlson Comorbidity Index	2.4 (1.9)	3.0 (2.0)	< .001
Coronary artery disease	156 (28.1)	31 (17.2)	.004
Hypertension	317 (57.0)	98 (54.7)	.595
With cancer	106(19.2)	62 (34.6)	< .001
Diabetes mellitus	254 (45.7)	73 (40.6)	.229
A history of stroke	111 (20.0)	26 (14.4)	.098
Chronic kidney disease	178 (32.0)	43 (23.9)	.039
**Initial clinical data**			
SIRS	2.3 (1.0)	2.4 (0.9)	.032
Quick-SOFA	1.5 (0.6)	1.7 (0.6)	.054
SOFA score	8.5 (3.5)	9.8 (4.0)	< .001
Respiration subscore	2.2 (1.2)	2.4 (1.2)	.054
Coagulation subscore	0.7 (1.0)	1.0 (1.2)	.002
Liver subscore	0.3 (0.7)	0.7 (1.1)	<.001
CV subscore	1.1 (1.6)	1.3 (1.6)	.075
CNS subscore	2.7 (1.2)	2.9 (1.1)	.118
Renal subscore	1.3 (1.4)	1.4 (1.4)	.533
White blood cell (count)	14,243.3 (8,033.9)	13,765.0 (8,786.0)	.498
Segmented neutrophil (count)	11,514.7 (7,154.9)	11,154.1 (7,644.9)	.564
Monocyte (count)	614.7 (538.2)	645.1 (783.2)	.630
Lymphocyte (count)	1,262.4 (1,398.6)	1,052.0 (1,200.4)	.071
C-reactive protein, mg/L	143.6 (114.5)	132.8 (114.7)	.334
Lactate, mmol/L	27.6 (25.9)	40.4 (34.8)	< .001
Procalcitonin, ng/mL	23.4 (47.3)	23.2 (47.8)	.975
$\Delta^{day3-day1}$ WBC (count)	-1,695.6 (7,167.3)	-181.1 (6,806.9)	.013
$\Delta^{day3-day1}$ seg (count)	-1394.4 (6,663.2)	-3.9 (6,366.9)	.014
$\Delta^{day3-day1}$ monocyte (count)	-15.3 (631.9)	-66.34 (725.9)	.365
$\Delta^{day3-day1}$ SeMo (%)	-4.6 (38.5)	3.9 (35.5)	.008

Table S2- Clinical characteristics of 727 sepsis patients stratified by the delta SeMo & WBC tool

Clinical characteristics	delta SeMo and WBC normal (N=275)	delta SeMo or WBC abnormal (N=324)	delta SeMo and WBC abnormal (N=128)	*P value*
Day 3				
SIRS	1.3 (0.9)	2.1 (1.0)	2.6 (0.8)	*< .001*
q-SOFA	1.3 0.5)	1.4 (0.6)	1.5 (0.6)	*.017*
SOFA scores	7.0 (2.9)	8.1 (3.6)	8.4 (4.1)	*< .001*
Respiration subscore	1.7 (1.0)	1.8 (1.0)	2.0 (1.0)	*.040*
Coagulation subscore	0.9 (1.1)	1.0 (1.2)	0.9 (1.2)	*.521*
Liver subscore	0.3 (0.8)	0.5 (0.9)	0.5 (0.9)	*.035*
CV subscore	0.3 (0.9)	0.7 (1.2)	0.9 (1.4)	*< .001*
CNS subscore	2.5 (1.0)	2.5 (1.1)	2.7 (1.0)	*.323*
Renal subscore	1.1 (1.4)	1.4 (1.6)	1.4 (1.5)	*.027*
White blood cell count	8,474.9 (2,011.6)	14,635.1 (7,385.8)	17,581.2 (6,677.7)	*< .001*
Segmented neutrophil count	6,593.0 (1,979.6)	12,213.2 (6,417.3)	15,496.1 (6,277.5)	*< .001*
Lymphocyte count	911.1 (519.5)	1,007.9 (993.8)	921.7 (593.9)	*.281*
Monocyte count	541.6 (398.8)	754.2 (840.0)	420.4 (281.5)	*< .001*
SeMo ratio	16.6 (13.5)	25.6 (28.7)	50.2 (30.6)	*< .001*
C-reactive protein, mg/L	101.4 (77.7)	133.6 (108.1)	159.3 (106.8)	*<.001*
Lactate, mmol/L	15.1 (9.4)	18.6 (11.7)	21.1 (13.3)	*<.001*
Procalcitonin	16.4 (39.1)	15.6 (31.9)	18.2 (38.5)	*.896*
Oxygenation index	5.8 (6.5)	7.3 (12.8)	7.8 (8.0)	*.118*
I/O, fluid balance	375.7 (1198.3)	579.3 (1336.3)	793.5 (1230.9)	*.007*

Table S3- Cytokine production of monocytes with and without LPS stimulation between the delta SeMo groups

**Immune status (N=144)**	**delta SeMo < 7**	**delta SeMo ≥ 7**	***P value***
**Day 3**			
LPS (-)	(N=105)	(N=39)	
G-CSF, pg/uL	19.6 (25.6)	21.0 (35.6)	*.784*
IL-10, pg/uL	4.2 (5.8)	3.7 (6.1)	*.668*
IL-1RA, pg/uL	18.3 (19.4)	11.2 (12.6)	*.034*
IL-1 β, pg/uL	6.4 (11.5)	5.2 (8.2)	*.544*
IL-6, pg/uL	170.1 (232.1)	95.3 (150.1)	*.064*
TNF- α, pg/uL	147.8 (155.0)	151.9 (377.9)	*.927*
VEGF, pg/uL	26.0 (23.6)	23.1 (22.2)	*.502*
LPS (+)	(N=104)	(N=39)	
G-CSF, pg/uL	60.3 (68.5)	78.4 (123.5)	*.389*
IL-10, pg/uL	9.0 (13.9)	10.9 (19.3)	*.527*
IL-1RA, pg/uL	24.4 (17.6)	28.1 (56.4)	*034*
IL-1 β, pg/uL	39.8 (95.3)	45.4 (119.0)	*.772*
IL-6, pg/uL	563.4 (618.7)	456.4 (386.7)	*.315*
TNF- α, pg/uL	435.2 (422.5)	417.1 (345.1)	*.811*
VEGF, pg/uL	42.5 (30.2)	31.6 (20.1)	*.014*

Peripheral blood mononuclear cells (1×10^6) were plated in a 5-mL round-bottom polystyrene test tube with 2 mL of sterile DMEM culture medium containing 1% heat-inactivated fetal bovine serum, 1 mM L-glutamine, and 1 mM sodium pyruvate. Inflammation was induced using LPS 100 ng/mL or not. The tubes were incubated at 37˚C in 5% CO_2 for 4 hours. Samples of the conditioned media were analyzed for cytokine expression levels.

Table S4- Correlation between delta SeMo, delta SOFA, delta WBC, and delta cytokine expression

	Trend Δd3-d1 HLA-DR%	Trend Δd3-d1 WBC count.	Trend Δd3-d1 SeMo	Trend Δd3-d1 SOFA
Day 1 WBC count.	.117 (148)	-.614** (727)	-.019 (727)	-.007 (727)
Day 3 WBC count.	.113 (148)	.310** (727)	.046 (727)	.088* (727)
Trend Δd3-d1 HLA-DR%	1 (148)	-.012 (148)	.023 (148)	.050 (148)
Trend Δd3-d1 WBC count.	-.012 (148)	1 (727)	.065 (727)	.092* (727)
Trend Δd3-d1 SeMo	.023 (148)	.065 (727)	1 (727)	.005 (727)
Trend Δd3-d1 SOFA	.050 (148)	.005 (727)	.005 (727)	1 (727)
Trend Δd3-d1 Seg count.	-.049 (148)	.919** (727)	.160** (727)	.081* (727)
Trend Δd3-d1 Lym count.	.157 (148)	.226** (727)	-.117** (727)	.025 (727)
Trend Δd3-d1 Mono count.	-.071 (148)	.378** (727)	-.326** (727)	.065 (727)
Trend d3/d1 G-CSF	-.018 (145)	.075 (151)	-.746** (151)	-.073 (151)
Trend d3/d1 IL-10	.018 (145)	.058 (151)	-.528** (151)	-.062 (151)
Trend d3/d1 IL-6	.120 (145)	.037 (151)	-.256** (151)	-.180* (151)

Data expressed as Pearson correlation (patient number with both tests)

*Correlation is significant at the 0.05 level; **Correlation is significant at the 0.001 level

Trend Δd3-d1: day 3 data minus day 1 data

Trend d3/d1: day 3 data divided by day 1 data

Table S5. The relationship between 28-day survivors and non-survivors among the delta of total mature granulocyte counts tool, immature granulocyte counts tool and the delta SeMo ratio & WBC count tool.

**28-day mortality**	**Survivor**	**Non-Survivor**	**p value**
Delta SeMo & WBC count tool			<0.001
Delta SeMo &WBC count normal	234 (85.1)	41 (14.9)	
Delta SeMo or WBC count abnormal	242 (74.7)	82 (25.3)	
Delta SeMo & WBC count abnormal	80 (62.5)	48 (27.5)	
Day 1 mature granulocytes (%)	n=549<br>98.8±4.9	n=168<br>98.1±3.3	0.970
Day 3 mature granulocytes (%)	n=541<br>98.5±4.0	n=166<br>97.9±3.9	0.100
Delta mature granulocytes (%)	n=535<br>0.4±5.5	n=164<br>-0.1±4.6	0.203
Day 1 immature granulocytes (%)	n=549<br>1.9±4.9	n=168<br>1.8±3.3	0.970
Day 3 immature granulocytes (%)	n=541<br>1.4±4.0	n=166<br>2.0±3.9	0.100
Delta immature granulocytes (%)	n=535<br>-0.4±5.5	n=164<br>0.1±4.6	0.203
Delta segmented-neutrophil count	n=541<br>-1236.0±6640.3	n=165<br>301.7±6278.0	0.009
Delta monocyte count	n=541<br>-2.1±633.0	n=165<br>301.7±6278.0	0. 301
Delta segmented neutrophil (≥0)	235(73.0)	87 (27.0)	0.044
Delta monocyte (≥0)	277 (77.2)	82(22.8)	0.669
